# A three-minute solid phase-based plant RNA extraction method

**DOI:** 10.1186/s43897-024-00084-5

**Published:** 2024-03-01

**Authors:** Guiling Liu, Gongfa Shi, Huijun Liu, Nuo Xu, Lijuan Fan, Ling Wang

**Affiliations:** College of Landscape Architecture, Northeast Forestry University, Harbin, 150040 China

Solid phase gene extraction (SPGE) was developed for in situ and rapid RNA extraction to enable the quantification of gene expression (Scherp and Hasenstein 2008). By inserting a pretreated probe directly into plant cells or tissues, it allowed fast RNA extraction that is suitable for spatiotemporal profiling of gene expression and fast screening of transformants during the process of developing genetically modified plants (Nestorova et al. 2017; Hasenstein et al. 2023). Because there is no homogenization that can release other biomolecules from plant cells and tissues, there is no need for purification prior to gene amplification. Thus, it can be used for plants rich in polysaccharides and polyphenolics. Major limitations of SPGE, however, include the high cost of the probe, long processing time, and short storage time of the probe. This significantly limits its application, calling for efforts to explore cost-effective probes and develop shorter and more convenient workflows. The research group implemented the technology proposed by Hasenstein et al. and made essential enhancements (Scherp and Hasenstein 2008).

*Iris sanguinea* Donn. ex Horn. is a cold-tolerant, summer-flowering plant native to northeastern China that grows in wetlands. *I. sanguinea* has great potential in gardening and the industry of cut flowers, as well as provides a natural resource to breed cold-resistant flower varieties. Thus, there is a growing interest in molecular studies in *I. sanguinea*, requiring cost-effective and fast extraction of mRNA.

We adopted the SPGE workflow (Fig. 1A) for the extraction of leaf RNA from *I. sanguinea*. The performance was evaluated by amplifying Actin-7 from the resulting mRNA. After reverse transcription and PCR, we found a positive band of Actin-7 (Fig. 1C), indicating successful extraction of mRNA using the SPGE method. We also used the modified CTAB method as a control here. A key improvement in the modified CTAB method was the addition of 3% of β-mercaptoethanol in the lysis buffer (2% CTAB, 2 M NaCl, 25 mM EDTA in 100 mM Tris, pH8.0) (Zhao et al. 2020). We found the resulting band was brighter than that of the SPGE technique. This was expected due to a higher RNA concentration from the modified CTAB method.

**Fig. 1:**
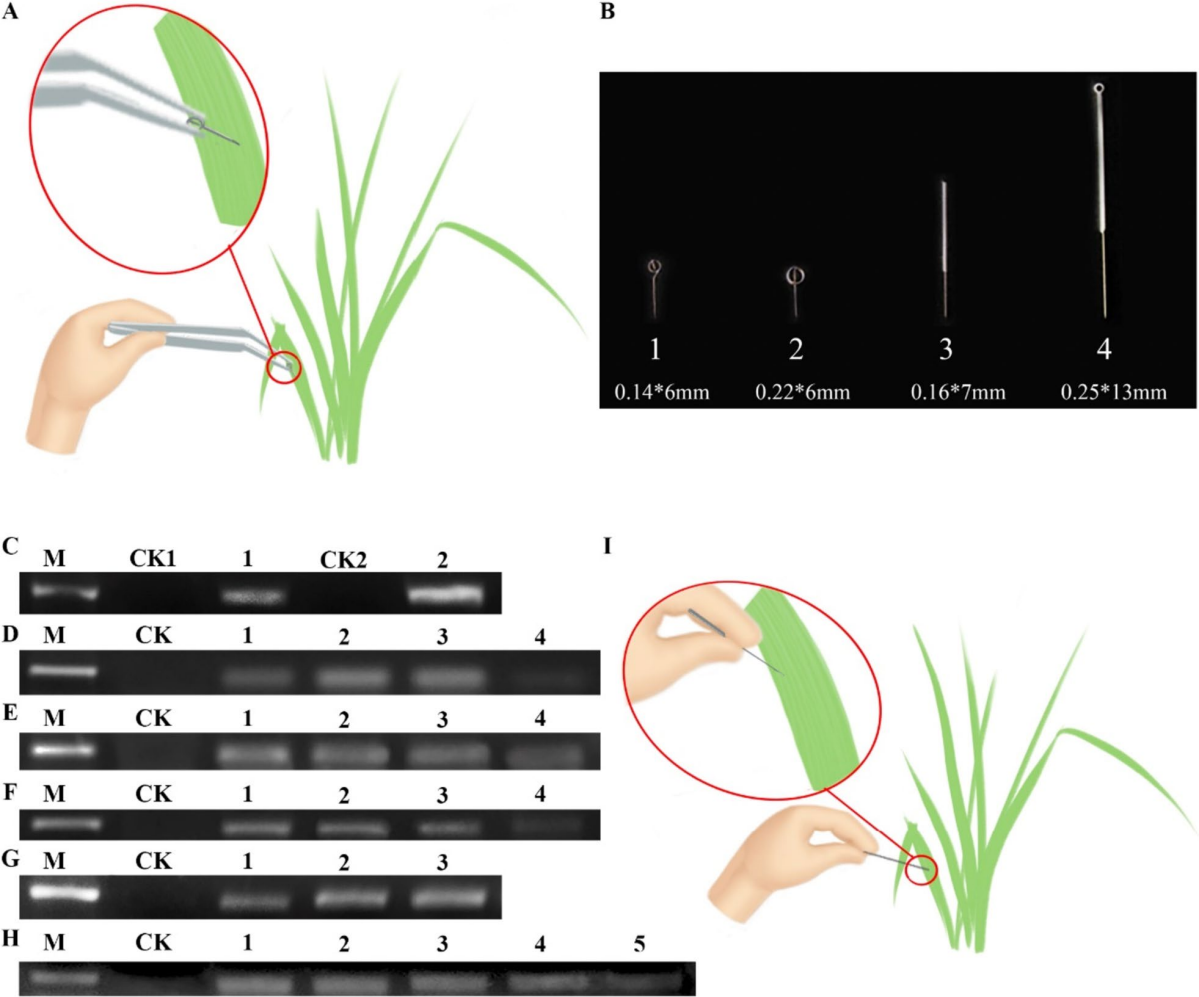
**A** Probe extracts RNA of* I. sanguinea.* **B** Probe of different specifications. **C** Evaluation of the SPGE method by PCR of Actin-7. M: DNA Marker 2000; CK1: negative control of SPGE technology; 1: Extraction of RNA by SPGE technology, reverse transcription. PCR product; CK2: negative control of improved CTAB method; 2: Extraction of RNA by improved CTAB method, reverse transcription, PCR product contrast. **D** Influence of different specification probes on the extraction quality of RNA, M: DNA Marker 2000; CK: negative control; 1: product of 0.14*6 mm probe; 2: product of 0.22*6 mm probe; 3: product of 0.16*7 mm probe; 4: product of 0.25*13 mm probe. **E** Influence of different culture time on the extraction quality of RNA. M: DNA Marker 2000; CK: negative control; 1: product of 12 h culture time; 2: product of 8 h culture time; 3: product of 4 h culture time; 4: product of 2 h culture time. **F** Influence of different embedding time on the extraction quality of RNA. M: DNA Marker 2000; CK: negative control; 1: product of 6 h embedding time; 2: product of 4 h embedding time; 3: product of 2 h embedding time; 4: product of 1 h embedding time. **G** Influence of different concentrations of Oligo dt25 on the extraction quality of RNA. M: DNA Marker 2000; CK: negative control; 1: product of 1uM Oligo dt25; 2: product of 3uM Oligo dt25; 3: product of 5uM Oligo dt25. **H** Influence of storage conditions on the storage time of the probe. M: DNA Marker 2000; CK: negative control; 1: product of 0d storage time; 2: product of 7d storage time; 3: product of 14d storage time; 4: product of 21d storage time; 5: product of 28d storage time. **I** A simplified gene extraction technique for RNA extraction in *I. sanguinea*

Encouraged by the successful implementation of SAGE in *I. sanguinea,* we next systematically optimized key parameters including probe size, incubation time, embedding time, oligo dt25, and storage conditions.

We first evaluated the impact of probe size on RNA quality. Four probes of different dimensions were tested (Fig. 1B). The result showed that probe 2 (0.14*6 mm) and 3 (0.16*7 mm) resulted in comparable PCR signals of Actin-7 (Fig. 1D), both of which were much higher than that of probe 1 (0.22*6 mm) and 4 (0.25*13 mm). Of note, the cost of probe 3 (0.0069$) is much lower than that of probe 2 (1.5$). In addition, the larger size of probe 3 made it possible to handle with hands directly without tweezers. Thus, probe 3 was used for the following experiments.

The length of the incubation between the probe and the solution is critical for high recovery of mRNA. Thus, we next determined the optimal incubation time using probe 3. We found that an increase in Actin-7 from 2 to 4 h incubation, but the signal was comparable among 4, 8, and 12 h (Fig. 1E). This indicated that the incubation time of the probe could be shortened to 4 h. For the embedding time, we tested 1, 2, 4, and 6 h and found that the later 3 embedding times resulted in similar levels of Actin-7 (Fig. 1F). To expedite the workflow, 2 h embedding time can be used.

In the next round of optimization, we tested the concentration of oligo dt25 because of its potential impact on the efficiency of the recovery of mRNA. Oligo dt25 of three concentrations (1, 3, and 5 µM) were tested and the two higher concentrations (3 and 5 µM) generated very comparable recovery of mRNA and thus amplification of Actin-7 (Fig. 1G). In later experiments, we used 3 µM of oligo dt25. To broaden the applications of SAGE, we next determined the shelf-life of the probe. We stored the probe for 7, 14, 21, and 28 days and compared the resulting mRNA to that of the fresh probe. We found that storage of the probe for 28 days still successfully led to successful extraction of mRNA and amplification of Actin-7 (Fig. 1H). In this study, the original solid-phase gene extraction technique was optimized to address the shortcomings of the technique, and mRNA was successfully extracted from petals and leaves using polysaccharide-polyphenol-rich Xizang plant material as samples, and the solid-phase extraction conditions were optimized (Fig. 1I).

The small amount of RNA extracted by solid-phase gene extraction technology has limited its application in gene cloning and molecular hybridization, but it has great potential for RNA quantification, gene expression studies in the spatial and temporal range of distribution and transgenic plant detection, and has significant superiority over previous functional gene extraction techniques, and its use can play a positive role in promoting transgenic breeding and germplasm innovation in ornamental plants.

## Data Availability

The authors confirm that all data in this study are included in this published article (and its Supplementary information file).
